# Play‐based groups for children with cerebral palsy and their parents: A qualitative interview study about the impact on mothers' well‐being

**DOI:** 10.1111/cch.12962

**Published:** 2022-02-01

**Authors:** Kirsten R. Prest, Aleksandra J. Borek, Anne‐Marie R. Boylan

**Affiliations:** ^1^ Nuffield Department of Primary Care Health Sciences University of Oxford Oxford UK

**Keywords:** cerebral palsy, child disability, groups, parents, social support, well‐being

## Abstract

**Background:**

Cerebral palsy (CP) is the most common childhood physical disability in developed countries. Parents of children with CP experience difficulties that can result in reduced well‐being. Health professionals supporting children with CP have been encouraged to focus on parental well‐being as this forms part of the child's essential environment. There is a lack of evidence about interventions that holistically support the whole family by providing therapeutic input for the child and support for parents. This study aimed to explore parents' experiences of play‐based groups for children with CP and their parents, with a focus on the groups' impact on parents' well‐being.

**Methods:**

Parents of children with CP who had attended play‐based groups in the year prior were recruited for this qualitative study. Semi‐structured interviews were conducted, audio‐recorded and transcribed verbatim. Participants' demographic characteristics were collected as contextual information. Data were analysed using an inductive thematic approach.

**Results:**

Ten mothers were interviewed. Overall, mothers had positive experiences of the groups and perceived them as an important influence on their well‐being. Four themes described mothers' experiences of the groups and the subsequent impact on their well‐being: (1) practical support, (2) connecting with others, (3) transitioning journeys and (4) different motivators, different experiences. Numerous factors influenced mothers' experiences of attending the groups and the subsequent impact on their well‐being. This included mothers' individual experiences of having a child with CP.

**Conclusions:**

Interventions combining practical and social support for the whole family can have a positive impact on the well‐being of mothers of children with CP. Care should be taken to provide individualised support for each family. There is no ‘one‐size‐fits‐all’ approach, and a package of care can provide multiple services that meet the varying needs of mothers and their children with CP.

**Key messages**

- Mothers of children with CP perceive the main benefits of attending the play‐based groups to be the support and training from multidisciplinary staff and therapists, connecting with other mothers in similar situations, and help in emotional transitions of acceptance of their child's disability. These seem to positively impact on most mothers' ability to cope and, thus, on their sense of well‐being.
- The well‐being of children with CP and their mothers is intricately linked and changes with time. Health professionals and services should take this into account particularly at key transition points, such as time after diagnosis.
- Allied health professionals' therapy input for children with CP can have a positive impact on mothers' well‐being.
- Peer support can have a positive influence on well‐being, so health professionals should facilitate connections between mothers in similar situations as part of supporting parents emotionally.
- Packages of service delivery informed by policy should be multifaceted as there is no ‘one‐size‐fits‐all’ approach for working with children with CP and their families.

## 1 INTRODUCTION

Cerebral palsy (CP) affects 2–2.5 of every 1000 live births and is the most common reason for a child with a physical disability in developed countries (National Institute for Health and Care Excellence, 2017). CP is an umbrella term used when describing injuries to the foetal or infant's brain that are irreversible but not progressive (National Institute for Health and Care Excellence, 2017). Systematic reviews on intervention strategies for children with CP have concluded that interventions should adhere to the International Classification of Functioning, Disability and Health: Children and Youth Version (ICF‐CY) (Hadders‐Algra et al., 2017; Morgan et al., 2016; Novak et al., 2013). The ICF‐CY emphasizes the shift from a narrow medical framework of disability to a biopsychosocial model, which focuses on children with CP and their parents more holistically by taking account of the environmental factors influencing the child (World Health Organisation, 2007). The ICF‐CY ideas have been adapted into a series of important concepts for children with CP, such as ‘family’, ‘function’ and ‘fun’ (Rosenbaum & Gorter, 2012). Play can be viewed as a child's primary occupation (Skard & Bundy, 2008). Children with CP may be limited in play due to restrictions in movement, sensory processing, cognitive abilities and their environment and social interactions (Blanche, 2008). Legislation recognizes that play and leisure opportunities for all children regardless of ability or disability is not only a right but essential for health and development (UNICEF United Kingdom, 1989; United Nations, 2007; World Health Organisation, 2015).

Parents of children with CP, particularly mothers, present with increased stress and depression levels, which have been attributed to reduced social support and self‐confidence, as well as their child's behaviour and level of impairment (Pousada et al., 2013). There is a need for specific and available interventions that aim to improve the well‐being of caregivers of children of CP (Irwin et al., 2019). In addition to the psychological health difficulties, greater frequencies of physical problems and chronic health conditions have been documented in this caregiver population. These have been related to the higher demand of care that their child's disability requires of them (Brehaut et al., 2004). As the child's immediate context is their family, it is assumed that their well‐being will be strongly influenced by the well‐being of their parents (Rosenbaum & Gorter, 2012).

Health professionals working with children with CP are therefore encouraged to focus on parent well‐being and to work collaboratively, as parents' experience and expertise need to be acknowledged (Hayles et al., 2015; Kruijsen‐Terpstra et al., 2016; Novak & Honan, 2019; Shevell et al., 2019). It has been emphasized that there is not a ‘one‐size‐fits‐all’ approach when working with families of children with CP (Hayles et al., 2015; Terwiel et al., 2017). It is therefore important to understand available interventions that are collaborative and tailored. Support groups, recommended by the National Institute for Health and Care Excellence (NICE) guidelines, are an example of an intervention that supports the well‐being of parents of children with CP (National Institute for Health and Care Excellence, 2017). Studies have found positive benefits for parents (Palit & Chatterjee, 2006) which may be linked to social support improving well‐being (Pousada et al., 2013).

**FIGURE 1:**
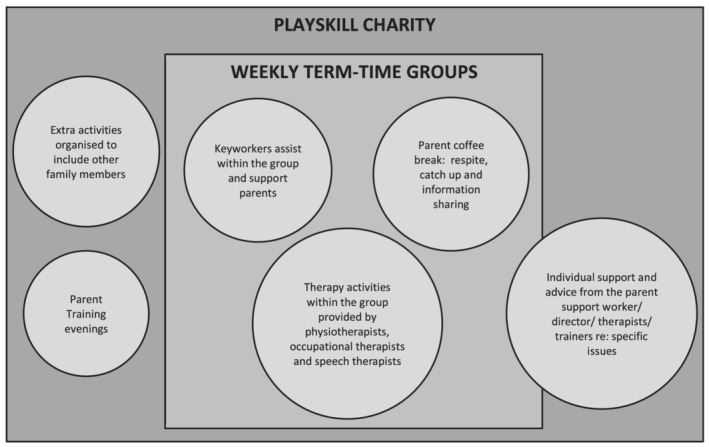
Package of support provided by Playskill charity

*Playskill* is a charity in the United Kingdom that runs play‐based groups for children with physical needs under the age of 6 years. They provide a package of support, including therapist‐run groups for children and parents, training workshops and individual advice/support for parents and extra events for the families. Various professionals are involved in supporting parents and children at Playskill including the director, the parent support worker, the therapists and the key workers (Figure 1). Playskill aligns with the ICF‐CY ideas as it focuses on fun and function for the children but also aims to foster social support between parents and provide practical advice and services, both of which contribute to parents' well‐being (Davis et al., 2009; Pousada et al., 2013). To our knowledge, there is currently paucity of research about the impact of groups that combine support for parents and therapy and play for the children with CP. This study therefore aimed to explore parents' experiences of attending these play‐based groups and their perceptions of the impact on parental well‐being.

## 2 METHODS

This was a qualitative study involving semi‐structured interviews with parents of children with CP who attended groups run by Playskill. The research is underpinned by a social constructionist paradigm that assumes that people's experiences and meaning are a result of societal dialogues, rather than being intrinsic to the individual (Burr, 1995). We note the influence of societal views and expectations of what disability is and what it means to be a parent or carer on the experience of having a child with an impairment (Shakespeare, 2006). As such, the findings from the study are therefore a reflection of parents' individual experiences within the context of the United Kingdom, cultural constructions of disability, social expectations of parenting, access to healthcare, schooling and financial support and views about friendship and social support. Patient and Public Involvement (PPI) took place in the form of three mothers of children with CP (not involved in Playskill but other similar groups) providing their insights and feedback on the research questions, interview guide and dissemination. The University of Oxford Research Ethics Committee approved the study (ref. R64920/RE001). Standards for Reporting Qualitative Research (SRQR) guidelines were used (Supporting Information).

The study was advertised to parents via email, posters and a presentation about the research given by the first author. Parents were invited to contact the researcher with expressions of interest. Participants were included in the study if they were the primary caregiver of a child with CP and attended Playskill groups in the year prior. All participants provided informed consent to participate.

Semi‐structured, face‐to‐face and telephone interviews were conducted between October 2019 and January 2020. Study‐relevant demographic information was collected at the start (Table 1). A semi‐structured interview guide (Appendix A) was developed based on the literature and discussions with mothers during PPI work. The guide was used flexibly to steer the interview and not to dictate its course. Priority was given to uncovering the individual experiences of each parent. Participants were reminded that their participation was voluntary, and they could withdraw from the study at any point. Interviews were audio‐recorded and then transcribed and anonymized by the researcher.

**TABLE 1:** Participant demographic information

Demographics of mothers and their children	*N* = 10
Mother's age, range (mean)	22–47 (35.9) years
Child's age, range (mean)	2–5 (3.5) years
Mother's marital status	
Married	6
Single	1
Co‐habiting with partner	3
Have other children	8
Type of CP	
Quadriplegia	3
Hemiplegia	3
Diplegia	1
Mixed	3
GMFCS^a^	
I	2
II	2
III	2
IV	2
V	2
Length of time attending groups, range (mean)	1–3.5 (1.9) years

^a^ GMFCS: Gross Motor Function Classification System records a child's functional level of movement (Palisano et al., 1997).

A thematic analysis approach was chosen due to its flexibility, compatibility with a variety of epistemological assumptions and the acknowledgement that the researcher plays an active role in identifying and reporting themes (Braun & Clarke, 2006). The initial data was coded inductively, line by line (without a pre‐existing framework). This formed a coding framework that was used to code remaining transcripts. New codes were added as analysis progressed, and previously coded transcripts were reanalysed. Similar codes were combined into categories. After the coding, themes were identified to address the research question and develop an insight going beyond the description of the data (Braun & Clarke, 2006). All coding was done by the first author, with the data, coding framework, themes and interpretations discussed between authors throughout the process.

## 3 RESULTS

Ten interviews were conducted. All participants were mothers with children between the ages of 2 and 5 (Table 1). Four themes were developed that captured perceptions of how the groups influenced parents' well‐being: (1) practical support, (2) connecting with others, (3) transitioning journeys and (4) different motivators, different experiences. Quotes are followed by pseudonyms and the number of years the parent attended the group.

### 3.1 Practical support

Practical support was perceived as one of the most helpful aspects of attending the groups. This involved the therapy for the children and the advice and support for parents.

All participants praised the therapy provided for their children by physiotherapists, occupational therapists and speech therapists. They found it helpful that their child was receiving frequent direct therapy but also that they were gaining ideas for therapeutic exercises to be completed at home.

It's been the most valuable thing that we have ever had for Jack … having these experts in the room once a week every single week and being with those other parents who are sort of along the same journey as you, you cannot put a price on that, it's amazing. Mary (1 year of attending the groups)

Participants valued the training workshops, which allowed for other family members to attend and learn too. The workshop topics that they found helpful included sensory processing, communication, reading and phonics, managing behaviours, postural management, self‐care skills and financial advice. Those who discussed the impact of understanding the financial support available to them (e.g. Disability Living Allowance) described how this information helped make their lives a little easier and provided new opportunities for them.

I think the training is very good and that's always been a big part of it. And in fact, I was told even before Ava started that there was a lot of involvement from parents. Susan (3.5)

Support for Education Health and Care Plans (EHCPs) was provided during training workshops and individually through the parent support worker. Participants described the EHCP process as ‘a minefield’, and how receiving support through the charity was one of the most valuable contributions to their own well‐being.

The EHCP process I found so stressful, and the parent support worker at Playskill, she did so much, she went to so many meetings, sent emails, did so much that I would never have been able to do. Cathy (3)

Signposting was another form of practical support discussed and valued. One mother described this practical support as having ‘a resource in your back pocket’ (Penny)—it was comforting to know that it was there even if not accessed.

### 3.2 Connecting with others

Another core aspect of the groups valued by the participants was the opportunity to connect with others in a similar situation, for example, within the groups, during the breaks, during organized events or meeting up with mothers outside the context of the groups.

Participants described feeling isolated. However, knowing others in similar situations with shared experiences helped make them feel understood and accepted. Some participants reported that they could talk to the parents that they met in the groups about topics that they could not speak to other friends and family about.

You need someone who's been in the same shoes, living on a different planet which we live on and they understand you, they know. So, there's just no other place where you can feel okay to go. Elizabeth (2)

Participants credited the groups for creating an environment where they felt less alone, specifically during the period just after the child's diagnosis when they experienced a lot of uncertainty and did not know others in the same situation. Connecting with others reduced those feelings of isolation.

… in the early phase [of attending the groups] it was massive because I did not have any kind of support for any of this stuff. So it was like a breath a fresh air really, to be able to speak to other parents that were dealing with the same things as you. Emma (1.5)

Mothers often felt that the groups created a space for them to connect and speak with each other in a way that supported their mental well‐being. Some described the group as a family because they became so comfortable with one another and relied heavily on the support. Other mothers did not form as close friendships, but still felt comfortable discussing relevant issues they were having. There was a general feeling of acceptance and that the space created was non‐judgemental.

One of the ways in which connecting with others helped parents to feel supported was through the sharing of skills and information with each other, which reportedly made a difference to participants' lives.

It's not only [about] the kids but it's the mums as well and there's loads of sharing of experience and equipment and especially about EHCPs, I learnt a lot from the other mums at different schools. So this is a two‐way kind of group for kids but for the parents as well. Elizabeth (2)

Another element of connecting was the relationships formed between the mothers and the key workers, many of whom also had children with various physical impairments. Due to such a deeply positive impact on participants, some said they would like to volunteer in the future for the charity to help others in similar situations.

Not all of the mothers felt connected and supported by others within the groups. Some identified barriers to forming connections, including a lack of confidence in opening up to others, not wanting to upset anyone, changing groups, turnover of parents and language barriers.

I feel like my English is not good enough to have a good conversation about my son you know I think this is the main problem. Because I can go anywhere, I can talk but I do not feel confident to talk about all the problems. Jenny (1)

So the first group that I was in there were a couple of mums that I really got on with, really sociable group, really chatty. This group I've only been in this term, they do not feel quite as close knit as we used to have in our group. But everyone is nice and friendly enough and we'll chat you know and share what's going on. Emma (1.5)

Other barriers to connecting with mothers included struggling to accept their child's disability, feeling ‘guilty’ because their child is not as needy as others and their situation being too sensitive to speak about.

Maybe [I do not feel supported by others because] it's just too raw and emotional in the different places that you are at. It's like sometimes when things are so personal you cannot share it. Melissa (1)

A suggestion to overcoming these barriers in connecting, particularly during the breaks, was to facilitate introductions between the mothers and give them an opportunity to explain their child's diagnoses to the group. Another participant suggested sending administrative information via email rather than during the breaks to allow more time for informal interaction.

Some parents are more confident than others, but it would just be [helpful] to have an introduction of why we are there and what conditions our children have. It might open up the group a bit, make everyone feel a bit more comfortable. Olivia (2)

### 3.3 Transitioning journeys

Participants' reports suggested that both children and mothers moved through personal transitions during their time attending the groups. These transitions influenced how the mothers viewed and experienced the groups, and it was also reflected in how they came to terms with their child's disability.

Participants attended the groups for various lengths of time. Those who had been attending longer felt that their child had gradually become more settled and comfortable and looked forward to the groups each week. Mothers discussed their child's attainment of targets very positively and enjoyed seeing their child proud of their own achievements, which improved mothers' sense of well‐being.

The whole reason you are there is for your child and if you are seeing positive things happening in their lives, that's got to have positive ramifications on you definitely. Melissa (1)

Just as the children progressed over time, mothers reported experiencing changes and transitions themselves. Although these transitions were individual and context dependent, some patterns were apparent. The mothers described emotional difficulties and challenges of the early days of coming to terms with their child's disability, especially when they compared them with children who were not disabled.

At first, I was a bit overwhelmed to go because I'd always gone to play groups when Alex had been the [only] unable child and was always sort of compared and I found that really, really tough especially in the first stages of diagnosis. Cathy (3)

When joining the groups, some described experiencing difficult feelings around their own situations when seeing children with more severe impairments within the group. Participants who knew others who had left the group felt that this was a contributing factor and that some parents' emotions were too painful and tender in the early stages of diagnosis.

When I first went there, not knowing what John would be able to do, seeing some of the children that do attend, just it can be a bit overwhelming (…) A previous parent that left, I know that she was finding it really hard to come to terms with it. She had previous children and then she had a little boy and she was finding it really hard to come to terms with it herself. And she did leave, so I think maybe that could be a reason why parents do not attend. Olivia (2)

Melissa described how she felt that other parents seemed to cope better than she did. She talked about wanting her child to be ‘normal’. She questioned if interacting with disability services like this charity was best and wondered if her child would be better served in mainstream education.

I felt like I was the only one ever crying and thinking everybody's coping really well; ‘you are all really smiley, you are all doing great. Why are you all doing so great because I feel like just completely devastated inside?’ Melissa (1)

Mothers who had been attending the group for longer discussed how the charity had supported them in their journeys of processing and adjusting to their child's diagnosis.

We're much more confident in each other, we have normalised her condition so that it's just Lucy, it's not because she's got cerebral palsy, it's because she's just Lucy. Penny (2)

### 3.4 Different motivators, different experiences

Participants found value in attending the groups, but their perceptions and motivations for attending varied, which influenced how they benefited from the groups. Some felt that the therapy for their child was the most valuable aspect of the group and therefore the main motivation for attending.

I suppose the main motivation [for attending] would have been to help Ava physically as we knew by that stage that she could not sit … So we knew that she needed more intervention than we were getting. Susan (3.5)

Others discussed how it was the only space for them to connect with others in a similar situation. This connection and support positively impacted their well‐being and was frequently a primary motivation for attending.

I think there's nowhere else that I've met people that are in similar situations to me and that alone is a support network … it's hard enough trying to deal with a diagnosis and when they first make it, you feel like you are completely alone because you do not know anybody else who is in the same situation. Until you go to a group like Playskill, and then you make your own family and a whole new group of friends. And that support helps your mental wellbeing so much. Cathy (3)

Some participants discussed how being signposted to services and receiving practical advice was one of the main benefits of attending the groups.

The various motivations for attending the groups depended on factors, such as how much emotional and practical support was available to them from family and friends, whether they were receiving extra privately funded therapy and their child's abilities and temperament.

I would not go there purely for the OT (Occupational Therapy) and PT (Physiotherapy) or Speech and Language because we do have some private sessions. So I'd say the support is the main thing for me. And Alana can go and be amongst kids where she does not need to compete. Elizabeth (2)

## 4 DISCUSSION

Barriers to parental well‐being in previous qualitative studies included a lack of access to information and health services, a lack of family support and financial barriers (Breitkreuz et al., 2014; Khanlou et al., 2017; Resch et al., 2010; Rodrigues et al., 2019). This study found that the play‐based groups and wider charity provided support in these areas and that the mothers who participated reported that attending helped their well‐being. Participants described how they felt supported by other mothers through sharing information about schools, equipment, resources and activity ideas. This is supported by previous qualitative and quantitative studies, which found that benefits of peer support groups included learning from each other's experiences, group problem‐solving and discussing everyday issues (Hayles et al., 2015; Kingsnorth et al., 2011; Law et al., 2002; Shilling et al., 2013). Another aspect of the peer support experienced in the groups was being able to connect with others in a similar situation. This is reinforced by previous literature that highlights the benefits of shared social identity experienced within similar group settings (Shilling et al., 2013) and the feeling of belonging (Solomon et al., 2001). There was some interest among the participants in volunteering at the charity in the future to support others. Studies have shown that reciprocity of support is an important aspect of social support among parents of children with complex needs (Kingsnorth et al., 2011; Law et al., 2002; Reid et al., 2011; Shilling et al., 2013; Solomon et al., 2001). One of the barriers to connecting with other parents within the groups was a lack of confidence. Healthcare professionals have a role to play in supporting parents' confidence, which has been associated with parental and child well‐being (Mas et al., 2019). One parent lacked confidence due to their English language ability, which demonstrates the importance of culturally and linguistically sensitive practices in this population. Utilizing interpreters or family members, accepting and embracing cultural differences and working together to find a way forward are all examples of how health professionals may provide culturally sensitive care in a similar population (Heer et al., 2016).

Parents in previous studies described how having a child with CP can be a continuous cycle of addressing a variety of new difficulties as their needs transform and develop over time (Hayles et al., 2015) and that parents' physical and emotional needs differ depending on the child's age (Park & Nam, 2019). This links to the transitioning journeys described by mothers in this study. The severity of the children's impairments in this study did not seem to link to participants' experiences of the group and their well‐being. Previous studies have not found clear correlations between the severity of a child's disability and parental well‐being (Manuel et al., 2003; Ones et al., 2005; Parkes et al., 2011; Skok et al., 2006). Rather, well‐being seems to be more dependent on perspectives on disability, which may change over time. A previous study focusing on support groups for parents of children with various impairments identified an overarching theme of identity change for parents (Solomon et al., 2001). Parents' needs, challenges, identities and well‐being therefore seem to transition with time. The groups seem to play an important role in supporting mothers with these transitions.

There was diversity in terms of ages, length attending the groups, the child's GMFCS level and type of CP, which allowed the development of a more nuanced understanding of the different motivations and experiences of the group. Rich narratives were elicited in the interviews in which participants shared a wide range of views that reflected the complexity of their experiences. The PPI activities at all phases ensured the study was acceptable, relevant and important to mothers. A limitation of the study was that parents who attended and left the groups were not interviewed as it was impossible to recruit them. Participants were asked why they thought others may have left the group in the past, which provided some insight. Only mothers expressed interest in volunteering for the study, reflecting the high proportion of mothers who attend Playskill. It would be beneficial for future research to include fathers, other family members or caregivers and those who discontinued from the groups.

Lincoln and Guba's (1985) techniques to enhance trustworthiness in qualitative research were used to ensure quality. *Credibility* was ensured through researcher triangulation, peer debriefing, prolonged engagement in the charity setting, member checking and PPI. An audit trail was used to document all phases of the study (*dependability and confirmability*), and thick description provided details about the context (*transferability*). Researchers practised *reflexivity* in examining their own position and how this impacted on decisions made throughout the process.

Further research could quantitatively assess and determine the impact of play‐based groups, and similar family‐centred services, on parents' well‐being and health. There are many cross‐sectional studies that have explored the well‐being of parents of children with CP. One of the findings of this study was that mothers' well‐being seems to transition over time. Future research should take a longitudinal approach to further explore how well‐being changes over time.

This study demonstrated that interventions that combine practical and social support for the whole family can have a positive impact on the well‐being of mothers of children with CP. It shows how health professionals' therapy input for the children can have a positive influence on mothers' well‐being. Facilitating connections among group participants is an important part of supporting mothers emotionally. There is no ‘one‐size‐fits‐all’ approach, and a package of care can provide multiple services that meet the varying and changing needs of mothers and their children with CP.

## CONFLICT OF INTEREST

None declared.

## ETHICS STATEMENT

This study received ethical approval from the University of Oxford Research Ethics Committee (ref. R64920/RE001).

## Supporting information




**APPENDIX 2:**Standards for Reporting Qualitative Research (SRQR) Checklist

## Data Availability

The data that support the findings of this study are available from the corresponding author upon reasonable request.

## APPENDIX A

### INTERVIEW TOPIC GUIDE

The following questions were used as a guide for the interviews but were not rigidly followed. Changes to this guide were made as the interviews progressed.

**Table:**

Topic	Example questions
Introductory	Thank them for taking part Explain that they are the expert of the experience of parenting a child with CP and understanding their well‐being, I am here to listen and try to understand Reassure that they are under no obligation to participate and can withdraw from the study at any point They are under no obligation to answer questions that they do not feel comfortable answering Reassure that everything that they say will be anonymous Check if it is okay to record
Being a parent of a child with CP	Can you tell me a bit about your child? Can you tell me about a typical day with your child? How do you and your child play? ○ What kind of things do you do together for fun? ○ When is your child most playful? What makes them laugh or smile? You said that you have other children *(if applicable)*, could you tell me about how your children may play together? ○ What type of activities do they do together? ○ What type of activities do you do all together as a family? ○ Where do you go to if you need to find information or answers to your questions about cerebral palsy?
The experience of the play‐based group	When and how did you first find out about the group? Why did you decide to join? Did you have any concerns or apprehensions about joining the group? Take me through how a typical group runs Can you tell me about the training that Playskill provides? Tell me what you enjoy about the group. Tell me about anything you may not enjoy ○ Why? ○ Can you think of an example when … How do you think your child finds the group? ○ What cues does your child give if they are enjoying or not enjoying the group?
Linking groups to well‐being	How does going to the group make you feel? What are your thoughts and feelings before you go? ○ Are you keen to go? ○ Do you have to drag yourself out the door? What motivates you to go? How do you feel during the group activities? Do you find it beneficial to you? ○ In what ways? Are there negative aspects to it? ○ Can you tell me about these? ○ How does that make you feel? Could you think of any changes to the groups that may positively impact your well‐being? ○ *Focus just on well‐being* Why do you think parents may choose not to attend the groups? Do you ever see parents outside of the group setting? ○ What sort of things do you do together? If you require support (emotional, physical, someone to talk to), who might you approach?
Concluding thoughts	How you feel that the well‐being of your family is linked to the well‐being of your child? ○ If so, could you explain how? Is there anything else that you would like to talk about regarding your experience of the groups and the impact on your well‐being? Thank them for their time

## References

[cch12962-bib-0001] Blanche, E. I. (2008). Play in Children with Cerebral Palsy: Doing With – Not Doing To. In L. D. Parham & L. S. Fazio (Eds.), Play in occupational therapy for children (pp. 375–393). Mosby Elsevier.

[cch12962-bib-0002] Braun, V. , & Clarke, V. (2006). Using thematic analysis in psychology. Qualitative Research in Psychology, 3(2), 77–101. 10.1191/1478088706qp063oa

[cch12962-bib-0003] Brehaut, J. C. , Kohen, D. E. , Raina, P. , Walter, S. D. , Russell, D. J. , Swinton, M. , O'Donnell, M. , & Rosenbaum, P. (2004). The health of primary caregivers of children with cerebral palsy: How does it compare with that of other Canadian caregivers? Pediatrics, 114(2), 182–191. 10.1542/peds.114.2.e182 15286255

[cch12962-bib-0004] Breitkreuz, R. , Wunderli, L. , Savage, A. , & McConnell, D. (2014). Rethinking resilience in families of children with disabilities: A socioecological approach. Community, Work & Family, 17(3), 346–365. 10.1080/13668803.2014.893228

[cch12962-bib-0005] Burr, V. (1995). An introduction to social constructionism. Routledge.

[cch12962-bib-0006] Davis, E. , Shelly, A. , Waters, E. , Boyd, R. , Cook, K. , & Davern, M. (2009). The impact of caring for a child with cerebral palsy: Quality of life for mothers and fathers. Child: Care, Health and Development, 36(1), 63–73. 10.1111/j.1365-2214.2009.00989.x 19702639

[cch12962-bib-0007] Hadders‐Algra, M. , Boxum, A. G. , Hielkema, T. , & Hamer, E. G. (2017). Effect of early intervention in infants at very high risk of cerebral palsy: A systematic review. Developmental Medicine and Child Neurology, 59(3), 246–258. 10.1111/dmcn.13331 27925172

[cch12962-bib-0008] Hayles, E. , Harvey, D. , Plummer, D. , & Jones, A. (2015). Parents' experiences of health Care for Their Children with cerebral palsy. Qualitative Health Research, 25(8), 1139–1154. 10.1177/1049732315570122 25711842

[cch12962-bib-0009] Heer, K. , Rose, J. , & Larkin, M. (2016). The challenges of providing culturally competent care within a disability focused team. Journal of Transcultural Nursing, 27(2), 109–116. 10.1177/1043659614526454 24857931

[cch12962-bib-0010] Irwin, L. , Jesmont, C. , & Basu, A. (2019). A systematic review and meta‐analysis of the effectiveness of interventions to improve psychological wellbeing in the parents of children with cerebral palsy. Research in Developmental Disabilities, 95, 103511. 10.1016/j.ridd.2019.103511 31670025

[cch12962-bib-0011] Khanlou, N. , Mustafa, N. , Vazquez, L. M. , Davidson, D. , & Yoshida, K. (2017). Mothering children with developmental disabilities: A critical perspective on health promotion. Health Care for Women International, 38(6), 613–634. 10.1080/07399332.2017.1296841 28278017

[cch12962-bib-0012] Kingsnorth, S. , Gall, C. , Beayni, S. , & Rigby, P. (2011). Parents as transition experts? Qualitative findings from a pilot parent‐led peer support group. Child: Care, Health and Development, 37(6), 833–840. 10.1111/j.1365-2214.2011.01294.x 22007983

[cch12962-bib-0013] Kruijsen‐Terpstra, A. , Verschuren, O. , Ketelaar, M. , Riedijk, L. , Gorter, J. W. , Jongmans, M. J. , & Boeije, H. (2016). Parents' experiences and needs regarding physical ^and^ occupational therapy for their young children with cerebral palsy. Research in Developmental Disabilities, 53‐54, 314–322. 10.1016/j.ridd.2016.02.012 26970858

[cch12962-bib-0014] Law, M. , King, S. , Stewart, D. , & King, G. (2002). The perceived effects of parent‐led support groups for parents of children with disabilities. Physical & Occupational Therapy in Pediatrics, 21(2–3), 29–48. 10.1080/J006v21n02_03 12029852

[cch12962-bib-0015] Lincoln, Y. S. , & Guba, E. G. (1985). Naturalistic Inquiry. Sage Publications.

[cch12962-bib-0016] Manuel, J. , Naughton, M. J. , Balkrishnan, R. , Smith, B. P. , & Koman, L. A. (2003). Stress and adaptation in mothers of children with cerebral palsy. Journal of Pediatric Psychology, 28(3), 197–201. 10.1093/jpepsy/jsg007 12654945

[cch12962-bib-0017] Mas, J. M. , Dunst, C. J. , Balcells‐Balcells, A. , Garcia‐Ventura, S. , Giné, C. , & Cañadas, M. (2019). Family‐centered practices and the parental well‐being of young children with disabilities and developmental delay. Research in Developmental Disabilities, 94, 103495. 10.1016/j.ridd.2019.103495 31499380

[cch12962-bib-0018] Morgan, C. , Darrah, J. , Gordon, A. M. , Harbourne, R. , Spittle, A. , Johnson, R. , & Fetters, L. (2016). Effectiveness of motor interventions in infants with cerebral palsy: A systematic review. Developmental Medicine and Child Neurology, 58(9), 900–909. 10.1111/dmcn.13105 27027732

[cch12962-bib-0019] National Institute for Health and Care Excellence . (2017). Cerebral palsy in under 25s: Assessment and management [NG62]. Accessed 13 March 2019. nice.org.uk/guidance/ng62 28151611

[cch12962-bib-0020] Novak, I. , & Honan, I. (2019). Effectiveness of paediatric occupational therapy for children with disabilities: A systematic review. Australian Occupational Therapy Journal, 66, 258–273. 10.1111/1440-1630.12573 30968419 PMC6850210

[cch12962-bib-0021] Novak, I. , Mcintyre, S. , Morgan, C. , Campbell, L. , Dark, L. , Morton, N. , Stumbles, E. , Wilson, S. A. , & Goldsmith, S. (2013). A systematic review of interventions for children with cerebral palsy: State of the evidence. Developmental Medicine and Child Neurology, 55(10), 885–910. 10.1111/dmcn.12246 23962350

[cch12962-bib-0022] Ones, K. , Yilmaz, E. , Cetinkaya, B. , & Caglar, N. (2005). Assessment of the quality of life of mothers of children with cerebral palsy (primary caregivers). Neurorehabilitation and Neural Repair, 19(3), 232–237. 10.1177/1545968305278857 16093414

[cch12962-bib-0023] Palisano, R. , Rosenbaum, P. , Walter, S. , Russell, D. , Wood, E. , & Galuppi, B. (1997). Development and reliability of a system to classify gross motor function in children with cerebral palsy. Developmental Medicine and Child Neurology, 39(4), 214–223. 10.1111/j.1469-8749.1997.tb07414 9183258

[cch12962-bib-0024] Palit, A. , & Chatterjee, A. (2006). Parent‐to‐parent counseling – A gateway for developing positive mental health for the parents of children that have cerebral palsy with multiple disabilities. International Journal of Rehabilitation Research, 29(4), 281–288. 10.1097/MRR.0b013e328010b9ad 17106343

[cch12962-bib-0025] Park, E.‐Y. , & Nam, S.‐J. (2019). Time burden of caring and depression among parents of individuals with cerebral palsy. Disability and Rehabilitation, 41(13), 1508–1513. 10.1080/09638288.2018.1432705 29378440

[cch12962-bib-0026] Parkes, J. , Caravale, B. , Marcelli, M. , Franco, F. , & Colver, A. (2011). Parenting stress and children with cerebral palsy: A European cross‐sectional survey. Developmental Medicine and Child Neurology, 53(9), 815–821. 10.1111/j.1469-8749.2011.04014.x 21707599

[cch12962-bib-0027] Pousada, M. , Guillamón, N. , Hernández‐Encuentra, E. , Muñoz, E. , Redolar, D. , Boixadós, M. , & Gómez‐Zúñiga, B. (2013). Impact of caring for a child with cerebral palsy on the quality of life of parents: A systematic review of the literature. Journal of Developmental and Physical Disabilities, 25(5), 545–577. 10.1007/s10882-013-9332-6

[cch12962-bib-0028] Reid, A. , Imrie, H. , Brouwer, E. , Clutton, S. , Evans, J. , Russell, D. , & Bartlett, D. (2011). ‘If I knew then what I know now’: Parents' reflections on raising a child with cerebral palsy. Physical & Occupational Therapy in Pediatrics, 31(2), 169–183. 10.3109/01942638.2010.540311 21189101

[cch12962-bib-0029] Resch, J. A. , Mireles, G. , Benz, M. R. , Grenwelge, C. , Peterson, R. , & Zhang, D. (2010). Giving parents a voice. Rehabilitation Psychology, 55(2), 139–150. 10.1037/a0019473 20496968

[cch12962-bib-0030] Rodrigues, S. A. , Fontanella, B. J. B. , de Avó, L. R. S. , Germano, C. M. R. , & Melo, D. G. (2019). A qualitative study about quality of life in Brazilian families with children who have severe or profound intellectual disability. Journal of Applied Research in Intellectual Disabilities, 32(2), 413–426. 10.1111/jar.12539 30353627

[cch12962-bib-0031] Rosenbaum, P. , & Gorter, J. W. (2012). The ‘F‐words’ in childhood disability: I swear this is how we should think. Child: Care, Health and Development, 38(4), 457–463. 10.1111/j.1365-2214.2011.01338 22040377

[cch12962-bib-0032] Shakespeare, T. (2006). The social model of disability. In L. Davis (Ed.), The disability studies reader (Vol. 2) (pp. 197–204). Routledge.

[cch12962-bib-0033] Shevell, M. , Oskoui, M. , Wood, E. , Kirton, A. , Van Rensburg, E. , Buckley, D. , Ng, P. , & Majnemer, A. (2019). Family‐centred health care for children with cerebral palsy. Developmental Medicine and Child Neurology, 61(1), 62–68. 10.1111/dmcn.14053 30294783

[cch12962-bib-0034] Shilling, V. , Morris, C. , Thompson‐Coon, J. , Ukoumunne, O. , Rogers, M. , & Logan, S. (2013). Peer support for parents of children with chronic disabling conditions: A systematic review of quantitative and qualitative studies. Developmental Medicine and Child Neurology, 55(7), 602–609. 10.1111/dmcn.12091 23421818

[cch12962-bib-0035] Skard, G. , & Bundy, A. C. (2008). Test of playfulness. In L. D. Parham & L. S. Fazio (Eds.), Play in occupational therapy for children (pp. 71–93). Mosby, Elsevier.

[cch12962-bib-0036] Skok, A. , Harvey, D. , & Reddihough, D. (2006). Perceived stress, perceived social support, and wellbeing among mothers of school‐aged children with cerebral palsy. Journal of Intellectual and Developmental Disability, 31(1), 53–57. 10.1080/13668250600561929 16766323

[cch12962-bib-0037] Solomon, M. , Pistrang, N. , & Barker, C. (2001). The benefits of mutual support groups for parents of children with disabilities. American Journal of Community Psychology, 29(1), 113–132. 10.1023/A:1005253514140 11439824

[cch12962-bib-0038] Terwiel, M. , Alsem, M. W. , Siebes, R. C. , Bieleman, K. , Verhoef, M. , & Ketelaar, M. (2017). Family‐centred service: Differences in what parents of children with cerebral palsy rate important. Child: Care, Health and Development, 43(5), 663–669. 10.1111/cch.12460 28326571

[cch12962-bib-0039] UNICEF United Kingdom . (1989). The UN convention on the rights of the child. Accessed April 1, 2019. https://www.unicef.org.uk/what-we-do/un-convention-child-rights/

[cch12962-bib-0040] United Nations . (2007). Convention on the rights of persons with disabilities. Accessed April 16, 2019. https://www.un.org/development/desa/disabilities/convention-on-the-rights-of-persons-with-disabilities.html 10.1515/9783110208856.203 18348362

[cch12962-bib-0041] World Health Organisation . (2007). International classification of functioning, disability and health: Children and youth version. Accessed 13 March 2019. https://www.who.int/classifications/icf/en/

[cch12962-bib-0042] World Health Organisation . (2015). The global strategy for women's, children's and adolescents' health, 2016–2030. Accessed April 16, 2019. https://www.who.int/life-course/partners/global-strategy/global-strategy-2016-2030/en/

